# The Joy and the Pain of Being Alone: Managing the Solitude-Loneliness (SOLO) Paradox in People High on the Autism Spectrum

**DOI:** 10.1007/s10567-026-00576-4

**Published:** 2026-06-16

**Authors:** Peter Muris, Anne Deckers, Franc Donkers, Thomas H. Ollendick, Sander Begeer

**Affiliations:** 1 Department of Clinical Psychological Science, Faculty of Psychology and Neuroscience, Maastricht University, P.O. Box 616, 6200 MD Maastricht, The Netherlands; 2 Stellenbosch University, Stellenbosch, South Africa; 3 Youz Maastricht Parnassia Group, Maastricht, The Netherlands; 4 Zuyderland Medical Center, Heerlen, The Netherlands; 5 Virginia Polytechnic Institute and State University, Blacksburg, USA; 6 Vrije Universiteit (VU) Amsterdam, Amsterdam, The Netherlands

**Keywords:** Autism, Autism spectrum disorder, Solitude, Loneliness, Solitude-loneliness (SOLO) paradox, Social connection, Hikikomori, Problematic internet use

## Abstract

This narrative review examines the complex relationship between autism and two fundamental social needs: the need to belong and the need to be alone, which underpin the conceptually distinct experiences of loneliness and solitude. After outlining core features of autism spectrum disorder−focusing on characteristic patterns of social functioning across the spectrum−we synthesize evidence showing that individuals with autism frequently report elevated loneliness alongside a comparatively stronger preference for solitude than typically developing peers. We conceptualize this seemingly counterintuitive pattern as the solitude-loneliness (SOLO) paradox. We propose that, within the autistic population, loneliness emerges from the dynamic interplay among social-communication differences, sensory hypersensitivity and related neurocognitive characteristics, coping strategies, and the chronic frustration of unmet social needs. Extending this framework, we explore how the SOLO paradox may, in some cases, contribute to more severe or maladaptive outcomes, including extreme social withdrawal (hikikomori) and problematic internet use. We conclude by discussing directions for future research, emphasizing the importance of translating conceptual advances into supportive strategies that meaningfully enhance the well-being of individuals on the autism spectrum.

## Introduction

The following are three responses given by autistic individuals when asked the question, “Do you ever feel lonely?”, which is part of the Autism Diagnostic Observation Schedule (Lord et al., 2012):

- 7-year-old male: “I don't feel lonely when I choose to be alone, but when people walk away and leave me by myself, then I feel lonely”
- 15-year-old female: “I love to do things on my own, but after 3 weeks of not meeting others, I start to feel lonely and really down”
- 23-year-old male: “I love spending time on my own, doing my own things in quiet solitude, but sometimes I need to be around other people and when no one is available, I feel lonely and sad”.^1^

All three responses compellingly illustrate a profound paradox often experienced by individuals with autism: although many of them, for a variety of neurocognitive, sensory, and social reasons, prefer or seek out solitude, this very preference can lead to feelings of loneliness and emotional distress. In other words, while they may find comfort in spending time alone, the resulting isolation may also be associated with feelings of disconnection and unhappiness. This tension between the desire for solitude and the innate human need for social connection is a defining and often painful aspect of life on the spectrum.

In this review, we begin with a brief overview of autism, with particular attention to characteristic patterns of social functioning across the spectrum. We then distinguish between loneliness and solitude, two conceptually distinct yet frequently conflated experiences that are central to human social life. Establishing this conceptual clarity allows us to examine how loneliness and solitude are uniquely experienced by autistic individuals, highlighting psychological, social, and environmental factors that shape these phenomena. Building on this foundation, we synthesize evidence showing that individuals on the autism spectrum often report elevated levels of loneliness, even while expressing a stronger preference for solitude compared to their typically developing peers. We conceptualize this apparent contradiction as the solitude-loneliness (SOLO) paradox, which reflects a complex interplay between autism-related social characteristics, individual coping strategies, and unmet social needs. We then extend this framework to consider more extreme or maladaptive outcomes potentially associated with the SOLO paradox in individuals high on the autism spectrum, including severe social withdrawal (hikikomori) and problematic internet use. Finally, we outline the practical implications of this framework and highlight key directions for future research, emphasizing the importance of translating theoretical insights into meaningful applications for people with autism while identifying open questions that warrant further empirical and conceptual attention.

## Autism and Social Functioning

Autism Spectrum Disorder (hereafter referred to as autism) is a neurodevelopmental condition characterized by differences in social communication and interaction, alongside restricted or repetitive patterns of behavior, interests, or sensory processing (American Psychiatric Association, 2022). Autistic traits are relatively common in the general population, with approximately 1% of individuals meeting diagnostic criteria for autism (Zeidan et al., 2022). Conceptualized as a spectrum, autism encompasses considerable heterogeneity in clinical presentation, support needs, and functional outcomes. Importantly, even individuals with milder or less overt presentations may experience enduring challenges in social participation, including forming and maintaining relationships, friendships, and supportive social networks (Tobin et al., 2014). Such difficulties can meaningfully affect quality of life across the lifespan (Ayres et al., 2018). Within this context, solitude and loneliness represent key constructs for understanding the social experiences of autistic individuals.

## The Concepts of loneliness and Solitude

When discussing the concepts of loneliness and solitude, it is important to consider two inherently opposing yet socially relevant human motivations: *the need to belong* and *the need to be alone*. The need to belong has been extensively described in the seminal work by Baumeister and Leary (1995), who argued that human beings possess “a pervasive drive to form and maintain at least a minimum quantity of lasting, positive, and significant interpersonal relationships” (p. 497). This drive is deeply rooted in attachment theory, which emphasizes the evolutionary importance of forming stable social bonds for survival and emotional regulation (Bowlby, 1982). According to Baumeister and Leary (1995), the need to belong incorporates two central aspects: (1) the need for frequent, affectively positive interactions with the same individuals, and (2) the need for these interactions to occur within a framework of long-term, stable caring and concern. Failures to satisfy either aspect can lead to a range of negative psychological and physical outcomes, with *loneliness* emerging as a predominant emotional response that reflects the individual’s experience of deficiencies in social relationships (Cacioppo & Patrick, 2008). Importantly, loneliness can be differentiated into two types: social loneliness, which arises from a shortage in the quantity of social contacts, and emotional loneliness, which stems from a lack of meaningful, intimate relationships – parallelling the two aspects of the need to belong described above (Weiss, 1973). As will be discussed later in this paper, several factors may frustrate the need to belong in individuals with autism, which may increase their vulnerability to both forms of loneliness. At the same time, it is important to acknowledge that many autistic individuals form deeply meaningful, loyal, and enduring relationships, highlighting the diversity of social experiences in this population (Finke, 2023; Yew et al., 2023).

In contrast to the need to belong, human beings also exhibit a fundamental need to be alone. Rather than constituting a rejection of social connectedness, solitude functions as its regulatory counterpart. This tendency reflects a desire to withdraw from others, both physically and digitally, to experience psychological freedom and to be oneself, temporarily disengaged from social norms, expectations, and the scrutiny or demands of others (Larson, 1990). When voluntarily chosen, solitude serves several adaptive functions, including psychological rest and recovery, opportunities for contemplation and spiritual experience, self-reflection, and the restauration of autonomy. It also enables engagement in personal leisure activities, creative or productive pursuits, and the maintenance of privacy and intimacy, thereby supporting meaningful engagement with both one’s inner and outer life (Long & Averill, 2003; Palgi et al., 2021). When this need is adequately satisfied, solitude is typically experienced as restorative and meaningful rather than distressing. However, just as frustration of the need to belong is associated with negative outcomes, the inability to attain desired solitude may lead to irritability, emotional exhaustion, and feelings of being overwhelmed (Coplan et al., 2019). Thus, the need to be alone does not negate the social nature of humans but instead represents a complementary motivational force that enables individuals to balance connectedness with self-regulation (Coplan et al., 2021). Notably, there are marked individual differences in both the need to belong and the need to be alone, as well as in how these needs are balanced. For some individuals, achieving a healthy or optimal equilibrium between connectedness and solitude can be particularly challenging, a difficulty that appears especially pronounced among people on the autism spectrum, as will be discussed in the following sections.

## Loneliness in Autism: Frustration of the Need to Belong

There has long been a tendency to characterize individuals with autism as having a reduced desire for social engagement, little to no social interest, or even an aversion to social stimuli (e.g., Kanner, 1943). This characterization has formed the foundation of the diminished social motivation theory, which posits that social interactions are inherently less rewarding for autistic individuals, leading to reduced attention to social cues and fewer efforts to initiate or maintain social relationships (Chevallier et al., 2012). According to this account, social difficulties in autism arise primarily from a lack of intrinsic motivation to engage with others. However, this core assumption has been increasingly challenged by first-person accounts and qualitative research involving individuals high on the autism spectrum, who frequently report a strong desire for social connection and meaningful relationships. These testimonies suggest that the issue is not diminished social motivation per se, but rather differences in how social interest is experienced and expressed (Jaswal & Akhtar, 2019). Consistent with this view, it has been argued that many individuals with autism share the same fundamental need to belong as their neurotypical peers, even if their social engagement takes unconventional or idiosyncratic forms (Dyer et al., 2025). For example, autistic individuals may seek connection through shared interests rather than small talk, prefer structured or one-on-one interactions over large social gatherings, or express affiliation through parallel activities rather than overt emotional signaling. Importantly, what counts as “typical” social behavior reflects culturally dominant interaction norms (e.g., small talk), which may not necessarily be inherently more functional forms of affiliation.

Building on this perspective, a growing body of evidence indicates that many autistic individuals do desire friendships and close interpersonal bonds, and want to become parents (Bauminger et al., 2008a, 2008b; Deckers et al., 2017; Sosnowy et al., 2019; Thom-Jones et al., 2025), even though the structure, expectations, and modes of engagement within these relationships may differ from those typically observed in neurotypical populations (Black et al., 2024; Jobe & Williams White, 2007; Kuo et al., 2013; Mendelson et al., 2016; Petrina et al., 2014; Thom-Jones et al., 2024). Autistic friendships are often organized around shared interests, predictability, and mutual understanding rather than frequent social contact or conventional markers of intimacy, yet they can be experienced as deeply meaningful and fulfilling (Cresswell et al., 2019; Lisboa White et al., 2024; Wu & Wang, 2025). Importantly, the prevalence of loneliness among individuals high on the autism spectrum further challenges the assumption of diminished social motivation. Multiple reviews have reported substantially elevated rates of loneliness in autistic populations, estimated to be approximately three to four times higher than in neurotypical comparison groups (for reviews, see Ee et al., 2019; Grace et al., 2022; Hymas et al., 2024), with evidence suggesting that loneliness in autism persists across the lifespan (Schiltz et al., 2024). These elevated levels of loneliness are accompanied by an increased risk for adverse emotional outcomes, including depression, self-harm, and suicidality (Hedley et al., 2018a, 2018b; Hymas et al., 2024). The experience of loneliness, by definition, presupposes an unmet desire for social connection, suggesting that social difficulties in autism are not rooted in a lack of interest in others, but rather in barriers to achieving desired forms of affiliation.

The latter is well illustrated in research conducted by Orsmond and colleagues (2004, 2013; Shattuck et al., 2011), who consistently demonstrated that social participation among adolescents and adults with autism is substantially lower than that of their typically developing peers. Autistic individuals meet friends less frequently, are seldom invited to social activities, and consequently experience high levels of social isolation. Differences in social interaction and communication appear to play a central role in this reduced participation: lower conversational experience and variations in social skills are associated with decreased social engagement. Pallathra et al. (2018) further specified how challenges faced by individuals on the autism spectrum – including atypical understanding of others’ mental states (theory of mind) and processing socio-emotional information (Velikonja et al., 2019), social skills challenges (Gates et al., 2023), and elevated social anxiety (Spain et al., 2018) – contribute to poorer functioning in social contexts. These difficulties limit the frequency and quality of social interactions and increase feelings of both social and emotional loneliness. Even when individuals with autism do engage socially, their relationships are often experienced as less meaningful, as differences in interests and cognitive styles can hinder mutual understanding with neurotypical peers (Quadt et al., 2024). Additionally, masking or camouflaging behaviors – defined as the suppression of autistic traits and the adaptation of one’s behavior to conform to neurotypical social norms (Cook et al., 2021) – may increase social acceptance to some extent. However, these strategies come at a psychological cost: reduced authenticity can negatively affect self-concept and increase vulnerability to what has been termed “autistic burnout” (Beck et al., 2020a, 2020b; Chapman et al., 2022; Raymaker et al., 2020; Scheeren et al., 2025).

While autistic individuals often experience difficulties engaging in social interactions – thereby increasing the risk of social isolation and, subsequently, loneliness – this challenge should not be understood solely in terms of individual impairments. Social participation is further complicated by the responses of others to the atypical social behaviors and idiosyncrasies associated with autism. In this context, Milton (2012) introduced the concept of the “double empathy problem”, which emphasizes that interactional breakdowns between autistic and non-autistic individuals are mutual rather than one-sided. There is evidence that autistic people are routinely perceived more negatively by their non-autistic peers, for example as less likeable and more awkward (Alkhaldi et al., 2021; Sasson et al., 2017; see Milton et al., 2022). Importantly, such negative perceptions do not remain merely attitudinal but may be translated into behavior, manifesting as ostracism – defined as the exclusion, rejection, or systematic ignoring (Williams, 2007) of individuals with autism – as well as more overt forms of mistreatment, including bullying (Maïano et al., 2016; Schroeder et al., 2014). These experiences of social marginalization appear to be associated with a reduced sense of belonging, increased distress, and heightened feelings of loneliness among autistic individuals (Cappadocia et al., 2012; Reinhard et al., 2020; Sebastian et al., 2009; Trimmer et al., 2017).

## Solitude Seeking in Autism: A Strong Need to be Alone

In the popular literature, autistic individuals have often been described as “hermits”, “loners”, or “lone wolves”, labels that imply a strong preference for solitude and an inherent need to be alone. Despite the pervasiveness of these characterizations, empirical research directly examining solitude seeking in autism remains extremely limited. Notable exceptions are the studies by Deckers et al., (2014, 2017), which examined the desire for social interaction in children and adolescents with autism. In their first experimental study (Deckers et al., 2014), 63 children high on the autism spectrum aged 8–12 years and 69 typically developing controls completed both an explicit self-report measure (the Wish for Social Interaction Scale) and an implicit behavioral measure (a computerized Face Turn Approach-Avoidance Task) assessing social approach tendencies. On the explicit measure, children with autism reported a significantly lower desire for social interaction compared to typically developing peers. In contrast, on the implicit task, children with autism showed a stronger tendency to pull social stimuli towards themselves, indicating heightened approach behavior and suggesting an intact, or even strong, need to belong.

The second study (Deckers et al., 2017), which used a survey-based design, replicated the explicit findings in a larger sample: children (7–11 years) and adolescents (12–18 years) with autism (*n* = 73) reported a lower explicit desire for social interaction than both clinically referred youths with attention-deficit/hyperactivity disorder (*n* = 76) and non-clinical controls (*n* = 106). Importantly, among autistic adolescents, the desire for social interaction increased with age and was accompanied by heightened feelings of loneliness. Taken together, these findings suggest that individuals with autism may exhibit a reduced explicit tendency to engage socially – consistent with a preference for solitude – while simultaneously demonstrating preserved or heightened implicit social approach motivation and increasing social needs across development. This dissociation indicates that solitude seeking in autism cannot be reduced to diminished social motivation alone but likely reflects more complex underlying mechanisms.

A central mechanism concerns the role of coping with social difficulties and social overwhelm. Social interactions often involve high cognitive, emotional, and perceptual demands for autistic individuals, including challenges related to social prediction, reciprocity, and rapid information processing (Lois-Mosquera et al., 2025). As a result, solitude may function as an avoidance-based coping strategy that reduces exposure to challenging social demands and protects against distress and fatigue (Neville et al., 2024; White & Roberson-Nay, 2009). Beyond immediate relief, solitude may also help conserve limited social and cognitive energy, allowing autistic individuals to allocate resources selectively toward social interactions that are more meaningful, supportive, or personally valued (Neville et al., 2024; Rosqvist et al., 2023). Closely related, solitude may also help buffer against social pain, such as feelings of rejection, exclusion, or repeated experiences of being misunderstood (Lois-Mosquera et al., 2025; Ren et al., 2016). In this sense, being alone may feel safer than social contexts, offering protection from misinterpretations, social errors, and negative interpersonal consequences that can arise during interaction. Another important factor concerns sensory processing. Many autistic individuals experience heightened sensory sensitivity (Baranek et al., 2006), and social environments (such as schools, workplaces, shopping malls, and public transport) are often rich in unpredictable sensory input (MacLennan et al., 2021). Solitude may therefore provide relief from sensory overstimulation by allowing greater control over the sensory environment and reducing cumulative sensory load (Neville et al., 2024; Quadt et al., 2024; Syu & Lin, 2018).

Beyond coping with external demands, solitude may also serve important identity-related functions. Qualitative works suggest that time-alone allows autistic individuals to “drop the mask” and fully be themselves, free from pressures to camouflage autistic traits or conform to neurotypical social norms (Grace et al., 2025; Lois-Mosquera et al., 2025). Solitude further enables deep immersion in personal interests and hobbies, which for people with autism are often described as intrinsically rewarding, regulating, and central to well-being (Long, 2025), but which may be difficult to pursue in social contexts that fragment attention or impose competing demands (Lizon et al., 2024). Finally, some autistic individuals insist on sameness, adhere strongly to routines and ritualized behavior patterns, and may show intense emotional reactions when these are disrupted (Bird et al., 2024; Lewis & Stevens, 2023; South & Rodgers, 2017). Solitude allows for greater control over routines and environmental demands, thereby increasing predictability and reducing intolerance of uncertainty, and as such may serve an important self-regulatory function.

Taken together, these mechanisms suggest that solitude seeking in autism reflects a multifaceted regulatory strategy rather than social withdrawal per se. By mitigating social, sensory, and emotional demands while supporting identity expression, predictability, and energy conservation, solitude may enable autistic individuals to regulate functioning in ways that are better aligned with their individual needs and capacities. However, the balance between the need for solitude and the need to belong is a delicate one, and a strong inclination toward being alone among individuals high on the autism spectrum may at times conflict with fundamental belonging needs, thereby increasing vulnerability to feelings of loneliness.

## The SOLO Paradox in Autism

**Fig. 1:**
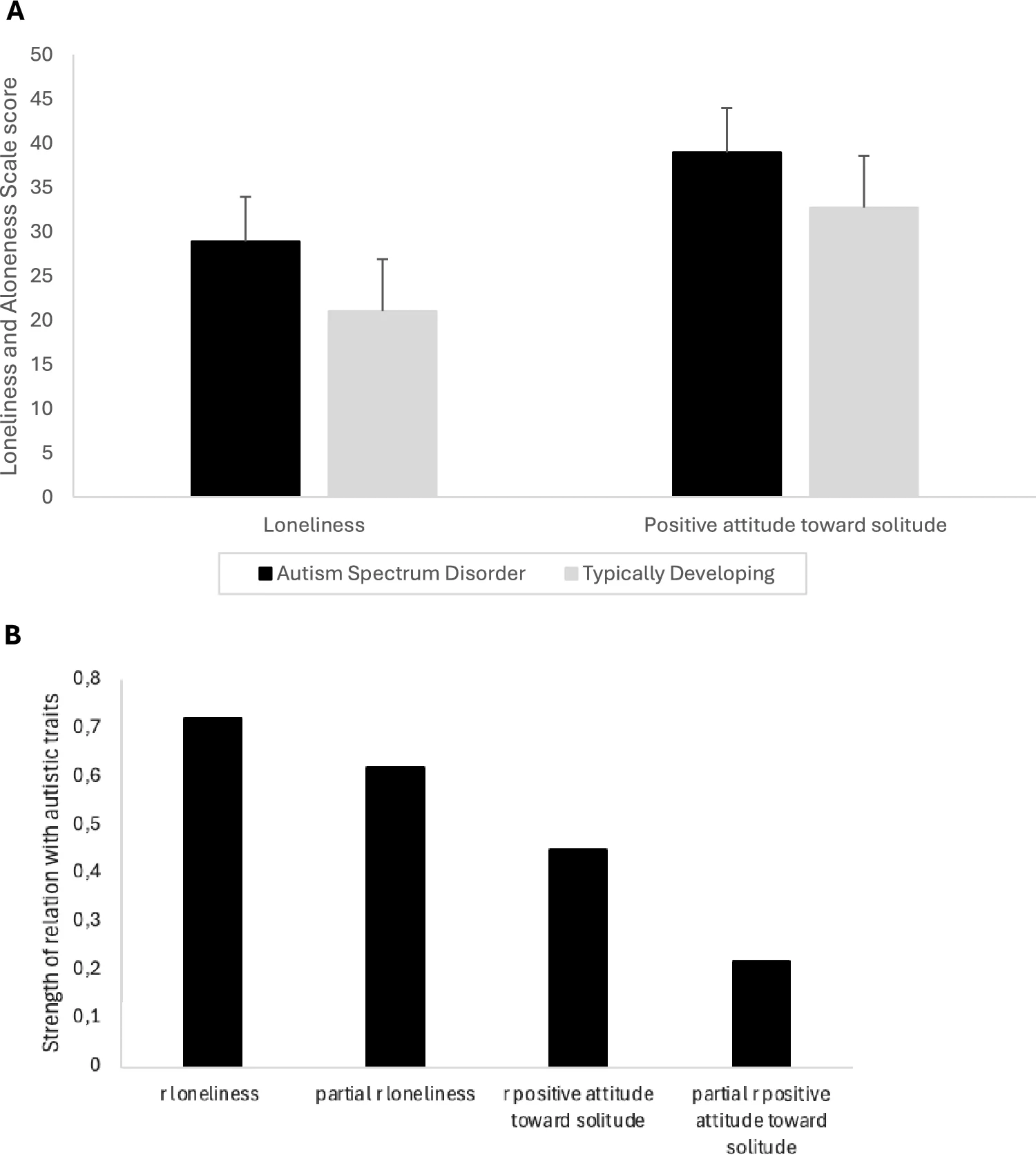
Association between autism and loneliness/positive attitude toward solitude. Panel A: Group differences in loneliness and positive attitude toward solitude scores between individuals with autism spectrum disorder (*n* = 64) and typically developing peers (*n* = 259). Panel B: Zero-order correlations and partial correlations between autistic traits and loneliness as well as positive attitude toward solitude, with partial correlations controlling for the alternate construct (*N* = 177). Based on: Muris et al. (2025a)

The previous sections indicate that individuals with autism, for various reasons, exhibit a strong need to alone, reflected in solitude-seeking behavior. At the same time, they often experience an unmet need to belong, which may lead to heightened feelings of loneliness. Data from a recent study by Muris et al. (2025a) provide a clear illustration of this SOLO-paradox. In this survey-based study, loneliness and positive attitudes toward solitude were assessed in a sample of adolescents and adults with and without autism, a subset of whom also completed a measure of autistic traits. As shown in Fig. 1, individuals with autism reported significantly higher levels of loneliness as well as a stronger positive attitude toward being alone compared to typically developing controls (Panel A). Moreover, autistic traits were positively correlated with both loneliness and a positive attitude toward solitude. Importantly, these associations remained statistically significant even after controlling for the complementary construct, indicating that autistic traits are independently associated with both elevated loneliness and a stronger preference for solitude (Panel B).

**Fig. 2:**
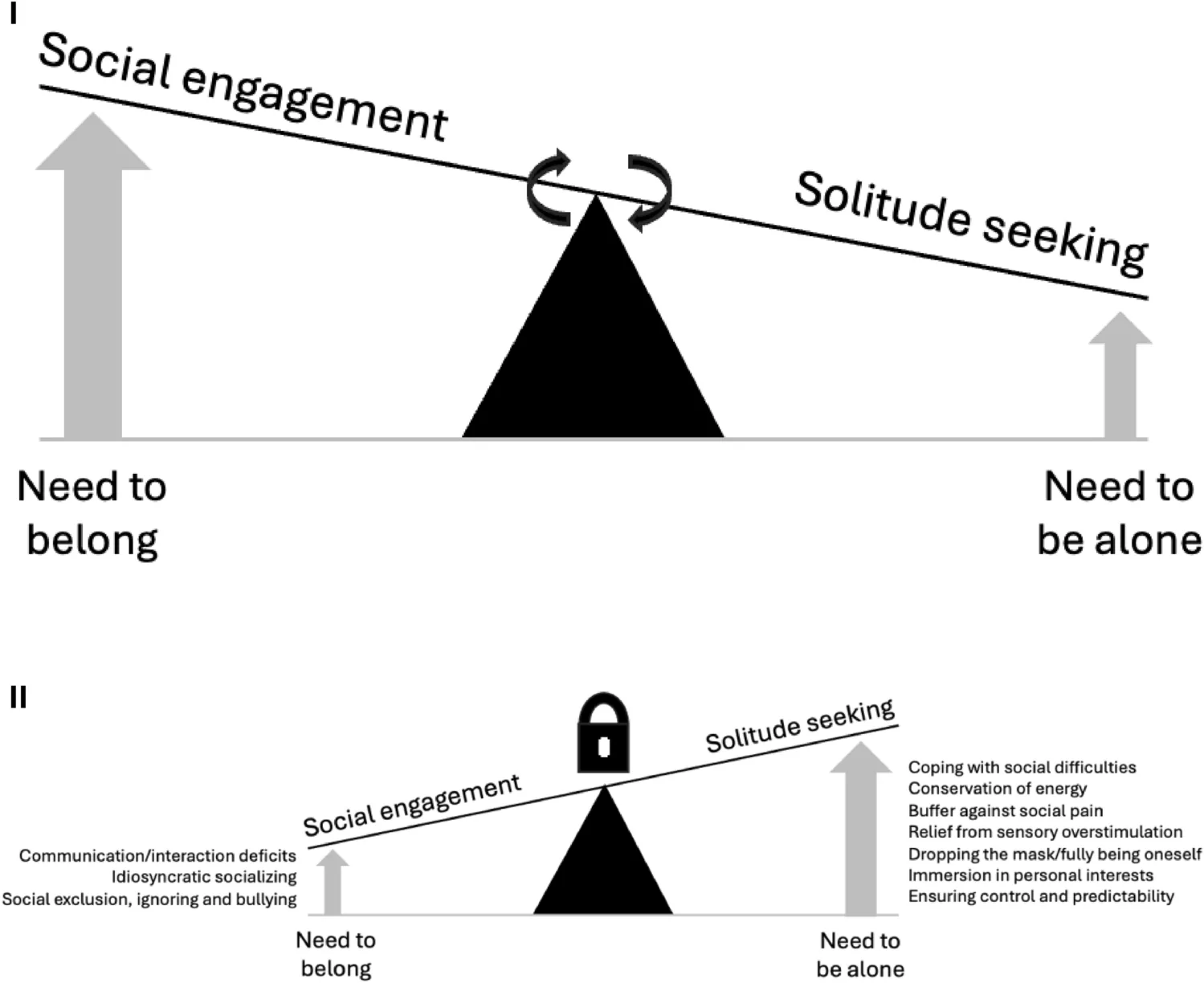
Dynamic interplay between the need to belong and the need to be alone and their influence on social behavior. Panel I: Archetypal scenario in which the need to belong is strong and flexibly regulated relative to the need to be alone. This dynamic balance is reflected in high levels of social engagement, with individuals actively seeking and generally succeeding in forming meaningful social connections, while also taking time for solitude when needed. Panel II: Hypothetical scenario characteristic of an individual high on the autism spectrum. An inflexible imbalance between the need to belong and the need to be alone is associated with reduced social engagement and increasing solitude seeking

Figure 2 illustrates the dynamic interplay between the need to belong and the need to be alone and their influence on social behavior. In the archetypal scenario (Panel I, see also Table 1), the need to belong is strong and expressed in high levels of social engagement, with individuals actively seeking and generally succeeding in forming meaningful social connections. At times, the need to be alone emerges, leading to solitude seeking, which supports mental rest, self-reflection, and renewed autonomy. Importantly, individuals in this archetypal pattern are able to satisfy both fundamental needs by flexibly alternating between social engagement and solitude, maintaining a healthy balance between connection and time alone.

**Table 1:** Profiles of social engagement, solitude seeking, and basic social need fulfillment in autism: Psychological consequences

Social engagement	Solitude seeking	Need to belong	Need to be alone	Social pattern	Outcome
High	Low	Fulfilled	Fulfilled	Highly affiliative*	Healthy social functioning
High	Flexible	Fulfilled	Fulfilled	Balanced engagement & autonomy^†^	Healthy social functioning
High	Low	Fulfilled	Unfulfilled	Over-engagement without autonomy*	Social exhaustion/burnout
High	Low	Unfulfilled	Fulfilled	Engaged but disconnected*	Emotional loneliness and psychological distress
Low but flexible	High	Fulfilled	Fulfilled	Autonomous low engagement*	Healthy social functioning
Low	High	Fulfilled	Fulfilled	Low engagement with fulfilled needs*	Contented solitude
Low	High	Unfulfilled	Fulfilled	Low engagement & unmet belonging/SOLO paradox*	Loneliness and psychological distress

^†^ Archetypal scenario. *Scenarios relevant for autism spectrum conditions

In autism (Fig. 2, Panel II), one possible pattern reflects a relatively inflexible imbalance between the need to belong and the need to be alone. In this scenario, autism-related characteristics – such as difficulties in social communication and interaction, idiosyncratic social behavior, and negative and excluding responses from others – may challenge the need to belong and contribute to reduced social engagement. At the same time, the need to be alone may be amplified by social exhaustion, sensory overload, anxiety, prior negative social experiences, and deep absorption in circumscribed interests, all of which increase the pull toward solitude. Over time, this interplay can become self-reinforcing and resistant to change: reduced social engagement limits opportunities to experience meaningful belonging, while increasing solitude further consolidates patterns of withdrawal. Importantly, the combination of low engagement and heightened solitude seeking is not inherently maladaptive for an autistic individual. When it aligns with a person’s social preferences and needs, it may reflect contented solitude – a state of fulfillment consistent with the prototypical personality profile described in autism (Chetcuti et al., 2021; Vuijk et al., 2018). Nevertheless, even contented solitude may pose challenges for daily functioning, including participation in school, work, or community contexts. However, when low social engagement and elevated solitude coexist with unmet belonging needs, the individual may experience loneliness and associated psychological distress, such as depression. This tension between a heightened pull toward solitude and an unfulfilled need to belong captures the SOLO paradox in autism.

At the same time, it is important to recognize that numerous other (dis)congruence patterns between social behaviors and fundamental social needs are possible, resulting in diverse social profiles and outcomes (see Table 1), many of which are commonly observed in the autistic population (Dubé et al., 2024; Scheeren et al., 2012, 2020; Wing & Gould, 1979). For example, an individual may display low engagement and high solitude seeking while retaining the ability to initiate social interactions when necessary. This configuration reflects an autonomous low-engagement profile, typically associated with adequate social functioning (Uljarevic et al., 2020). Conversely, some autistic individuals may exhibit high social engagement combined with low solitude seeking. In certain cases, individuals may engage extensively with others – either in person or online – while camouflaging their autistic traits, leaving their need for solitude chronically unmet. Over time, this imbalance can result in social exhaustion: despite appearing socially competent, they experience substantial internal fatigue and stress (Mantzalas et al., 2022). In other instances, high levels of engagement may occur in atypical or unconventional ways, such as through distinctive communication styles or behaviors that constrain reciprocal connection. Despite frequent social interaction, these individuals may experience emotional loneliness, characterized by feelings of disconnection and a diminished sense of belonging (Verity et al., 2025).

**Fig. 3:**
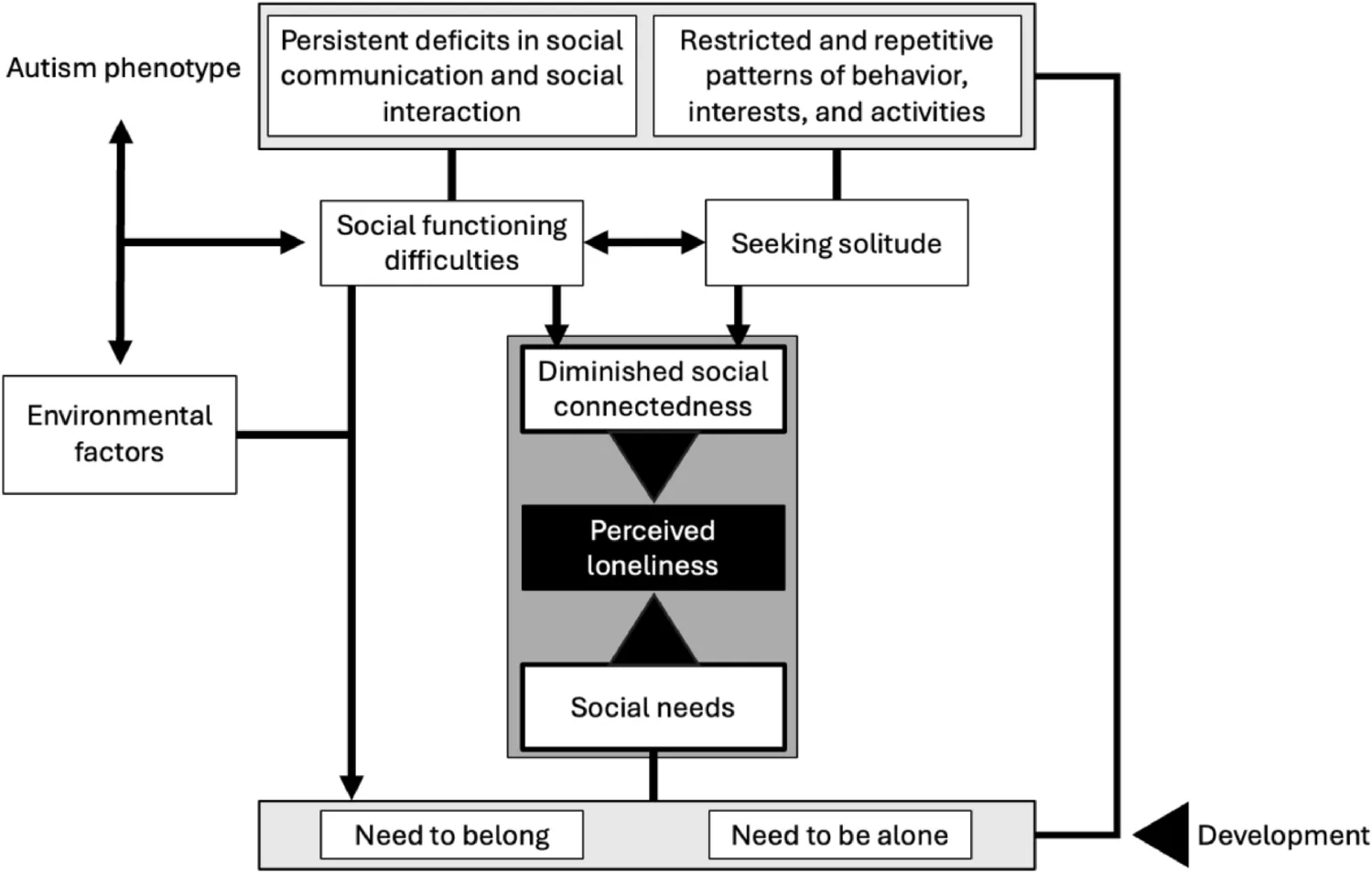
The SOLO paradox: Misalignment between social needs and social connectedness in autism spectrum conditions

In sum, the SOLO paradox should be understood as one – albeit frequently occurring – configuration within the broader spectrum of social need-behavior patterns in autism. As illustrated in Fig. 3, core phenotypic characteristics of autism (i.e., persistent differences in social communication and interaction, alongside restricted and repetitive patterns of behavior and interests), in interaction with environmental factors (e.g., stigma, victimization, sensory overload, social misunderstanding, structural exclusion), may contribute to difficulties in social functioning and increased tendency to seek solitude. In the long term, these processes may become mutually reinforcing. This dynamic can ultimately result in diminished social connectedness and, in its wake, the subjective experience of perceived loneliness, which emerges from the interplay between fundamental social needs – most notably the need to belong and the need to be alone. The figure highlights how these needs can become misaligned: when the need to belong remains insufficiently met while the pull toward solitude is strong, loneliness may arise despite a genuine preference for being alone.

Importantly, it is necessary to recognize that fundamental social needs, such as the need to belong and the need to be alone, should not be understood as wholly fixed, innate, or entirely autonomous preferences. Rather, these needs may be profoundly shaped by environmental experiences and broader social contexts. For autistic individuals in particular, experiences such as chronic social pressure, repeated interpersonal difficulties, and persistent stigma may gradually alter how social connection is experienced and desired. Over time, such experiences may diminish the individual’s motivation or capacity to seek belonging, while simultaneously increasing the appeal, comfort, or perceived safety of solitude. From this perspective, a preference for being alone should not always be interpreted a fully conscious, freely chosen, or stable disposition. In many cases, it may instead reflect an adaptive response to the social environment the autistic person has repeatedly encountered. Put differently, the need for belonging and the inclination toward solitude may emerge not only from intrinsic preference, but also from cumulative relational experiences and the emotional consequences of navigating environments that feel invalidating, exhausting, or unsafe.

This line of reasoning is somewhat analogues to findings within the camouflaging literature. Research on autistic camouflaging suggests that masking behaviors are not always the result of deliberate or strategic decision-making. Rather, camouflaging may become partially unconscious, deeply embedded within autistic people’s lived experiences, and shaped by ongoing attempts to secure social acceptance, avoid rejection, or maintain safety in predominantly non-autistic environments (Cage & Troxell-Whitman, 2019). In this sense, camouflaging can function as a survival-oriented coping mechanism that develops through repeated interaction with social norms and expectations. Similarly, preferences for social withdrawal or solitude may sometimes reflect adaptive processes shaped by environmental demands and accumulated social experiences, rather than purely autonomous personal choice.

Notably, the salience of the SOLO paradox is likely to vary across development. It may become particularly pronounced during adolescence, a developmental period in which establishing and maintaining meaningful peer relationships constitutes a central developmental task (Brown & Larson, 2009). Because peer group inclusion and social reciprocity gain heightened importance during this stage – and are often more difficult to achieve for autistic youth – the discrepancy between desired belonging and actual connectedness may widen. At the same time, many autistic adolescents may continue to display elevated solitude seeking, driven by social exhaustion, sensory sensitivities, and a sustained preference for solitary activities. This continued pull toward solitude may further complicate efforts to achieve peer integration, thereby intensifying the tension between belonging needs and social behavior. As a result, adolescence may represent a window of increased vulnerability to loneliness and associated psychological distress in autism (Deckers et al., 2017), amplifying the experiential core of the SOLO paradox.

## Adverse Correlates of the SOLO Paradox

While the SOLO paradox captures a dynamic tension between the need to belong and the need to be alone, this imbalance does not unfold in a social vacuum. In some individuals, particularly during adolescence and emerging adulthood, the self-reinforcing cycle of reduced engagement and heightened solitude seeking may crystallize into more pervasive patterns of withdrawal. In its more extreme manifestations, such withdrawal can resemble phenomena such as hikikomori, a condition characterized by prolonged social isolation and confinement to the home (Kato et al., 2019), or may co-occur with excessive or maladaptive engagement in digital environments (Weinstein & Lejoyeux, 2010). These patterns are not merely comorbid features but may instead represent behavioral expressions of a sustained misalignment between fundamental social needs and lived social experience. When offline belonging remains chronically elusive and solitude becomes both refuge and default mode of regulation, online contexts may emerge as alternative spaces in which connection, autonomy, and control feel more attainable (e.g., Davis, 2001). From this perspective, hikikomori and problematic internet use can be understood as contextualized correlates of the SOLO paradox – adaptive in intent, yet potentially maladaptive in outcome – arising when the tension between belonging and solitude is negotiated primarily through withdrawal and digital immersion.

Within this framework, increasing attention has been directed toward the role of autism as a vulnerability context in which such dynamics may become particularly pronounced. Emerging evidence suggests an overrepresentation of autistic traits and formal autism diagnoses among individuals exhibiting hikikomori-like withdrawal (e.g., Brosnan & Gavin, 2023; Muris et al., 2025a). Core characteristics of autism – including social communication differences, heightened sensory sensitivity, and a preference for predictability – may intensify the subjective costs of social participation, especially in adolescence when peer norms become more complex and socially demanding (Steinberg, 2023). Repeated experiences of misunderstanding, exclusion, or social exhaustion can amplify the appeal of controlled, low-demand environments such as the home (see Reinhard et al., 2020). In this sense, hikikomori among autistic individuals may not reflect a rejection of sociality per se, but rather a self-protective attempt to regulate overwhelming social input in contexts where belonging feels chronically unattainable or unsafe (Dell’Osso et al., 2025; Muris & Ollendick, 2023). The SOLO paradox may therefore be particularly salient in autism: the need for meaningful connection persists, yet the social environments in which connection is typically structured may be misaligned with neurodivergent social styles. Importantly, a fulfilling social life for an autistic individual may not mirror neurotypical norms, yet they can still cultivate satisfying relationships, pursue valued activities, and thrive in diverse social and professional contexts.

A parallel pattern can be observed in the domain of problematic internet use among individuals with autism. Digital environments often reduce the ambiguity and immediacy of face-to-face interaction, allow greater control over self-presentation, and provide access to interest-based communities that validate specialized passions – features that can be especially appealing for autistic youth and young adults (Muris et al., 2025b; Murray et al., 2022; Normand et al., 2022). For some, online spaces may facilitate authentic connection and identity exploration in ways that offline settings do not (Carpita et al., 2024; Kim & Bottema-Beutel, 2019). However, when digital engagement becomes the primary or exclusive arena for social fulfillment, it may inadvertently reinforce offline withdrawal and limit opportunities to develop broader relational competencies (Muris et al., 2025c). In the context of the SOLO paradox, problematic internet use in autism can thus be conceptualized not merely as impulsive overuse, but as a compensatory regulatory strategy – a means of satisfying belonging needs while preserving autonomy and minimizing social overload (Finkenauer et al., 2012). Over time, such compensatory reliance may deepen the imbalance between solitude and connection, entrenching a cycle in which digital immersion substitutes for, rather than complements, diversified social participation.

In contemporary societies marked by increasing digitalization, remote participation, and the proliferation of home-delivered services, the structural conditions for negotiating belonging and solitude have shifted profoundly. Opportunities to meet social needs online while minimizing face-to-face demands have become more accessible than ever, potentially intensifying the tension at the heart of the SOLO paradox. For many individuals, this environment may heighten the risk that solitude becomes the path of least resistance when belonging feels effortful or uncertain (Reimann, 2021). Yet this balancing act may be particularly complex for individuals with autism or elevated autistic traits. Given heightened sensitivity to social overload and greater reliance on controlled environments for regulation, they may be more vulnerable to becoming entrapped in patterns such as hikikomori-like withdrawal or problematic internet use. In such cases, strategies initially adopted to manage misalignment between social needs and social contexts may ultimately exacerbate that misalignment, deepening rather than resolving the SOLO paradox. These dynamics highlight the importance of interventions for individuals with autism, serving the goal of supporting meaningful connection while preserving autonomy and well-being.

## Practical Implications: Interventions Targeting the SOLO Paradox in Autism

When considering our model depicting the core dynamics of the SOLO paradox (Fig. 3), at least three avenues for clinical intervention emerge. First, interventions may focus on enhancing autistic individuals’ opportunities for successful social connection by strengthening social skills. There is abundant evidence that structured social skills trainings are effective in supporting children, adolescents, and adults with autism (Babb et al., 2020; Dubreucq et al., 2022; Gates et al., 2017). These programs help participants acquire concrete, knowledge-based scripts that support more confident navigation of social situations (e.g., Laugeson et al., 2012). Typically, these programs rely on explicit, didactic instruction targeting specific domains of social functioning – such as asking for help, declining a request, initiating conversations, or responding to negative behavior from others. Such approaches – delivered either face-to-face or through technology – can meaningfully increase social understanding and behavioral repertoire (e.g., Soares et al., 2021). However, the transfer of these explicitly learned skills to complex, real-life interactions is sometimes limited (Begeer et al., 2015; Nejati et al., 2024). In response, more performance-based and naturalistic interventions have been developed and increasingly evaluated (e.g., MacDonald et al., 2022). These approaches emphasize implicit learning through spontaneous peer interactions embedded in enjoyable, structured group activities within a supportive environment, and they tend to show stronger generalization effects. At the same time, a critical caveat is warranted: many social skills programs implicitly train normative interaction styles typically displayed by non-autistic individuals (Lerner et al., 2023). As such, they may encourage camouflaging of autistic traits. Although camouflaging can facilitate social acceptance and connectedness, it may also come at a substantial energetic cost, increasing the risk of social exhaustion and burnout (Nel et al., 2025). From the perspective of the SOLO paradox, social skills interventions are therefore not inherently problematic, but their “dosage” and framing are crucial. Social engagements should preferably be time-limited, predictable, and conducted in small, manageable groups, with explicit attention to recovery needs and authenticity.

Second, interventions may target the regulatory functions of solitude by addressing anxiety and broader difficulties in emotion regulation. As noted earlier, solitude is not inherently maladaptive; it can serve restorative, self-regulatory, or interest-driven purposes. Concerns arise, however, when solitude becomes predominantly avoidance-based – serving as the sole or primary strategy for managing overwhelming anxiety or social uncertainty (Newson et al., 2003; O’Nions & Eaton, 2020). In such cases, interventions that reduce anxiety and strenghten adaptive emotion regulation capacities are warranted. Cognitive-behavioral therapies have demonstrated efficacy in reducing anxiety symptoms and emotional dysregulation among autistic individuals, although effect sizes tend to be somewhat smaller than those observed in typically developing populations (Beck et al., 2020a, 2020b; Blackwell et al., 2025; Menezes et al., 2022; Rosenau et al., 2024; Ung et al., 2015). Importantly, recent developments have emphasized adapted and co-designed interventions that explicitly incorporate autistic perspectives, sensory sensitivities, and characteristic cognitive styles (Cullingham et al., 2024). Crucially, the aim of such approaches is not to eliminate solitude as a legitimate and sometimes necessary coping strategy, but to prevent it from becoming rigid, fear-driven, and socially constricting. Clinically, the objective is to broaden the individual’s repertoire of regulatory strategies – enhancing flexibility and choice – rather than pathologizing a preference for time alone.

Third, interventions may focus more directly on fostering belongingness. Beyond skill acquisition and anxiety-distress reduction, the SOLO paradox underscores the fundamental human need to feel accepted and valued. Efforts to enhance belonging may include peer-mentoring models (Siew et al., 2017), social prescribing (Featherstone et al., 2022), interest-based affinity groups – whether in-person or online (Chen et al., 2021; Stolba et al., 2024), inclusive educational and workplace practices (Failla et al., 2025; Petersson-Bloom & Holmqvist, 2022), family-based interventions that strengthen mutual understanding (Pennant, 2025). Furthermore, emerging evidence suggests that online environments may provide a comparatively accessible context for autistic individuals to initiate and maintain contact, as they reduce the demands associated with real-time interpretation of tone of voice, facial expression, and body language (Kuo et al., 2014; Van Schalkwyk et al., 2017). In this respect, social media can represent a viable and sometimes empowering route to belonging. At the same time, some scholars have cautioned that exclusively remote forms of socializing may not always foster the same depth of interpersonal bonding as face-to-face interaction (Baumeister et al., 2026), highlighting the importance of a balanced and individualized approach.

Finally, it is important to emphasize that belongingness is not achieved solely by increasing the quantity of social interactions, but by improving their quality – ensuring that interactions are characterized by reciprocity, respect, and shared meaning (Maitland et al., 2021). From this perspective, intervention targets extend beyond the autistic individual to the broader social environment. Promoting neurodiversity-affirmative attitudes (Morrison et al., 2025; Wagland et al., 2025), reducing stigma, and educating peers and professionals (Davidson & Morales, 2023; Kim et al., 2024) may be equally critical components. Clinically, the aim is to help autistic individuals find or build supportive social contexts in which authenticity is possible and connection does not depend on sustained camouflaging (Hull et al., 2017). In this way, belongingness can function as a protective factor, buffering against loneliness without undermining the restorative value of chosen solitude.

## Directions for Future Research

Besides the practical implications, there are several promising lines of inquiry for advancing research on the SOLO paradox. A central priority for future research is the systematic assessment of social needs in autistic individuals, particularly the need to belong and the need to be alone. While early theoretical accounts proposed diminished social motivation in autism (Chevallier et al., 2012), recent research has questioned this view (e.g., Lindsay-Webb et al., 2026), and empirical work directly comparing these needs with those of typically developing peers is still scarce (see for an exception: Deckers et al., 2017). Studies employing validated instruments such as the Need to Belong Scale (Leary et al., 2013) and the Preference for Solitude Scale (Burger, 1995) would allow researchers to disentangle whether individuals high on the autism spectrum display a distinct motivational profile characterized by coexisting high needs for both connection and aloneness. Importantly, involving autistic individuals in the development of such studies – for instance through participatory or co-production approaches (Den Houting et al., 2021) – could help ensure that the constructs, measures, and interpretations adequately capture autistic experiences of social motivation and solitude.

A second priority is the need for more empirical demonstrations of the SOLO paradox, particularly across the autism spectrum. Notably, much of the existing research disproportionately focuses on autistic individuals without intellectual disability, potentially limiting generalizability (Taylor et al., 2025). It is therefore essential to examine whether similar processes operate among individuals with co-occurring intellectual disability or higher support needs. Research should explicitly test the co-occurrence of heightened loneliness and stronger solitude preference and determine whether this pattern is especially pronounced among individuals with higher levels of autistic traits. Such work would clarify whether the paradox reflects a robust psychological phenomenon.

Third, a more nuanced understanding of the motives underlying solitude seeking is essential. Solitude may arise from negative motives (e.g., social anxiety or interpersonal discomfort) or positive motives (e.g., enjoyment of quiet or self-reflection). The Motives for Solitude Scale (Thomas & Azmitia, 2019) offers a promising tool to differentiate between these pathways, although the Experience Sampling Method could also be useful for capturing momentary motivations (Chen et al., 2014). Determining whether solitude is primarily avoidant or intrinsically motivated in autism would help refine interpretations of the paradox.

Fourth, future studies should directly test whether loneliness arises from an imbalance between experienced social connectedness and individual social needs. A person-environment fit framework may help determine whether discrepancies, rather than absolute levels of contact, predict loneliness (Klein & Macoun, 2025). Longitudinal designs are especially critical, particularly during adolescence – a developmental stage marked by heightened peer salience and neurobiological change (Lisboa White et al., 2024). It would also be preferable to examine multiple dimensions of loneliness (i.e., emotional, social, and existential; Van Tilburg, 2021).

Finally, insights from the SOLO paradox should inform interventions that are ideally co-designed with autistic individuals (Cullingham et al., 2024) to better align social engagement, solitude seeking, and individual needs. Such approaches should adopt a neuroinclusive perspective, recognizing that wellbeing is not determined by achieving a prescribed number of social connections, but by supporting personally meaningful and sustainable patterns of social interaction and alone time. Accordingly, interventions should aim to reduce loneliness and social stress without pathologizing adaptive forms of solitude, instead emphasizing flexibility, autonomy, and respect for neurodiverse patterns of social motivation. Importantly, responsibility for fostering meaningful connection should not rest solely on autistic individuals through efforts to improve social skills, emotion regulation, and anxiety management. Rather, interventions should also encourage non-autistic peers, families, educators, and communities to actively scaffold opportunities for authentic and mutually meaningful relationships in everyday life.

## Conclusion

As Long and Averill (2003) observed, “People have biological needs for attachment, affiliation, and sociality, yet they incidentally seek to spend time in solitude” (p. 22). Solitude can serve adaptive and restorative functions, supporting rest, recovery, and contemplation (Nguyen & Rodriguez, 2024). However, solitude is not always sought for these positive purposes alone. In some cases, it may also emerge as a response to social strain, sensory overload, or repeated experiences of interpersonal difficulty. It is within this more ambivalent landscape of solitude – as both restorative and defensive – that the present review is situated.

Specifically, individuals with autism appear to experience a distinctive imbalance. Despite expressing a stronger preference for solitude than their typically developing peers, they report heightened levels of loneliness. We conceptualize this apparent contradiction as the SOLO paradox. Rather than reflecting inconsistency, the SOLO paradox captures a dynamic tension shaped by autism-related social communication differences, sensory sensitivities, personal coping strategies, and persistent unmet needs for meaningful connection. For many autistic individuals, solitude may function both as a refuge and as a risk – offering protection from overwhelming social demands while, paradoxically, intensifying feelings of isolation.

The SOLO paradox thus underscores a central complexity of the autistic social experience: the desire for connection does not vanish in the presence of solitude, and the choice of solitude does not negate the need to belong. Instead, solitude and loneliness can coexist in ways that reflect adaptation, self-protection, and longing all at once. Recognizing this coexistence allows us to move beyond simplistic interpretations and toward a more nuanced understanding of how individuals with autism navigate the delicate balance between autonomy and affiliation.

## Data Availability

No datasets were generated or analysed during the current study.

## Footnotes

1 These statements are derived from autistic individuals and have been anonymized and paraphrased to protect participant confidentiality.
